# The neuroprotective effects of melatonin against diabetic neuropathy: A systematic review of non-clinical studies

**DOI:** 10.3389/fphar.2022.984499

**Published:** 2022-08-31

**Authors:** Asieh Hosseini, Mahedeh Samadi, Maryam Baeeri, Mahban Rahimifard, Hamed Haghi-Aminjan

**Affiliations:** ^1^ Razi Drug Research Center, Iran University of Medical Sciences, Tehran, Iran; ^2^ Neuroscience Research Center, Iran University of Medical Science, Tehran, Iran; ^3^ Toxicology and Diseases Group (TDG), Pharmaceutical Sciences Research Center (PSRC), Tehran University of Medical Sciences (TUMS), Tehran, Iran; ^4^ Pharmaceutical Sciences Research Center, Ardabil University of Medical Sciences, Ardabil, Iran

**Keywords:** diabetic neuropathy, melatonin, oxidative stress, inflammation, systemic review

## Abstract

**Backgrounds:** Diabetes can cause diabetic neuropathy (DN), a nerve injury. High blood sugar (glucose) levels can harm nerves all over your body. The nerves in your legs and feet are the most commonly affected by DN. The purpose of this study was to conduct a review of melatonin’s potential neuroprotective properties against DN.

**Method:** A full systematic search was conducted in several electronic databases (Scopus, PubMed, and Web of Science) up to March 2022 under the PRISMA guidelines. Forty-seven studies were screened using predefined inclusion and exclusion criteria. Finally, the current systematic review included nine publications that met the inclusion criteria.

**Result:** According to *in vivo* findings, melatonin treatment reduces DN *via* inhibition of oxidative stress and inflammatory pathways. However, compared to the diabetes groups alone, melatonin treatment exhibited an anti-oxidant trend. According to other research, DN also significantly produces biochemical alterations in neuron cells/tissues. Additionally, histological alterations in neuron tissue following DN were detected.

**Conclusion:** Nonetheless, in the majority of cases, these diabetes-induced biochemical and histological alterations were reversed when melatonin was administered. It is worth noting that the administration of melatonin ameliorates the neuropathy caused by diabetes. Melatonin exerts these neuroprotective effects *via* various anti-oxidant, anti-inflammatory, and other mechanisms.

## 1 Introduction

Diabetic neuropathy (DN) is one of the most prevalent and debilitating complications of diabetes mellitus, severely limiting the patient’s quality of life and imposing a significant burden on the healthcare system (Leinninger et al., 2006). The global prevalence of diabetes mellitus has been steadily increasing, with an estimated 9.3% (463 million people) in 2019, expected to increase to 10.9% (700 million people) by 2045 (Saeedi et al., 2019). The DN prevalence in diabetic patients varies from 9.6 to 88.7% worldwide. This variation may be due to age, diabetes types, glucose control, disease duration, and available health facilities (Amour et al., 2019).

Despite DN’s widespread prevalence and devastating effects, there is still no treatment to prevent its progression. The current management of DN is based on three principles: intensive blood sugar control, pathogenetic treatments, symptomatic therapy, which does not possess sufficient efficacy (Hosseini and Abdollahi, 2013). Therefore, designing effective drugs against DN based on novel therapeutic strategy and targets is essential. Current experimental strategies for treating or preventing DN inhibit several metabolic pathways involved in its development, such as controlling oxidative stress and reducing chronic inflammation (Saberi Firouzi et al., 2018; Sarvestani et al., 2018; Galeshkalami et al., 2019). Therefore, administering anti-oxidants seems to be one of the most sensible treatment approaches for DN, and some studies have confirmed this hypothesis (Hosseini et al., 2010; Hosseini et al., 2011; Ghaznavi et al., 2015; Najafi et al., 2015).

Melatonin (N-acetyl-5-methoxytryptamine) is an endogenous neurohormone derived from tryptophan and produced by the pineal gland. Melatonin is a well-known powerful anti-oxidant (Nishida, 2005). It is an effective scavenger of various free radicals, and also it up-regulates the expression of anti-oxidant enzymes and down-regulates inflammatory protein expression in all cells (Rodriguez et al., 2004; Jung et al., 2010). Eliminating free radicals by melatonin is the primary mechanism for counteracting neurotoxicity caused by oxidative stress, making it a potent neuroprotective (Nishida, 2005). As reported in several literature reviews, melatonin reduces diabetic complications by neutralizing oxidative damage (Afifi, 2013; Zephy et al., 2015; Mok et al., 2019). In addition, several documents from *in vitro* and *in vivo* studies demonstrate potent neuroprotective effects of melatonin against DN (Srinivasan et al., 2006; Arreola-Espino et al., 2007; Negi et al., 2010; Zencirci et al., 2010).

In the present systematic review, we aimed to evaluate and discuss the possible protective role of melatonin against DN *in vivo*. In addition, it was tried to answer the following questions: (i) the underlying mechanisms of DN, (ii) the role of melatonin against DN-induced neurotoxicity, (iii) and the underlying mechanisms of the preventive role of melatonin in DN. To ultimately achieve the above aims, we conducted a comprehensive literature review to analyze the protective role of melatonin in DN.

## 2 Methods

A systematic search was conducted in this investigation, adhering to the Preferred Reporting Items for Systematic Reviews and Meta-Analyses (PRISMA) guideline (Moher et al., 2009). Additionally, a PICO framework was used (Moher et al., 2009). This framework includes participants (P): DN (*in vitro* studies) and/or patients/animals with diabetes-induced neuropathy adverse effects (clinical/*in vivo* studies); intervention (I): patients/animals/cells with DN; comparison (C): patients/animals/cells treated with melatonin; and outcomes (O): there were two critical outcomes: 1) changes in the neuropathy following diabetes treatment compared to control/untreated groups and 2) changes in the DN.

### 2.1 Search strategy

We conducted a thorough systematic search to identify all relevant works on “the role of melatonin in diabetic-induced neuropathy” in both medical subject heading (MeSH) and advance in the electronic databases Scopus, PubMed, and Web of Science up to March 2022, using the keywords of melatonin AND “Diabetic Neuropathy” OR “Diabetic Autonomic Neuropathy” OR “Diabetic Neuropathies” OR “Diabetic Autonomic Neuropathies” OR “Diabetic Neuralgias” OR “Diabetic Neuralgia” OR “Painful Diabetic Neuropathy” OR “Painful Diabetic Neuropathies” OR “Symmetric Diabetic Proximal Motor Neuropathy” OR “Asymmetric Diabetic Proximal Motor Neuropathy” OR “Diabetic Asymmetric Polyneuropathies” OR “Diabetic Asymmetric Polyneuropathy” OR “Diabetic Mononeuropathy” OR “Diabetic Mononeuropathies” OR “Diabetic Mononeuropathy Simplices” OR “Diabetic Mononeuropathy Simplex” OR “Diabetic Amyotrophy” OR “Diabetic Polyneuropathy” OR “Diabetic Amyotrophies” OR “Diabetic Polyneuropathies” in title, abstract or keywords.

### 2.2 Process of study selection

The inclusion criteria for this systematic review were full-text scientific papers that met the following criteria: 1) they were written in English; 2) they focused on the role of melatonin in diabetes-induced neuropathy (as defined by the keywords mentioned above); 3) they contained adequate findings; 4) there were no restrictions on publications that included clinical, *in-vitro*, or *in-vivo* studies; and 5) there were no restrictions on publication year. Additionally, this study excluded 1) hemodynamic data, 2) unrelated publications, 3) review articles, 4) case reports, 5) book chapters, 6) oral presentations, 7) letters to the editors, 8) posters, and 9) editorials.

### 2.3 Process of data extraction

Two researchers examined each eligible study and then extracted the following data: a) author name and year of publication, b) models (tissue) and duration, c) diabetes-induced agent (dosage) and route of administration, d) outcomes of DN impairment, e) melatonin dosage & route of administration/duration of administration, and f) melatonin administration outcomes.

## 3 Results

### 3.1 Literature search and screening

**FIGURE 1:**
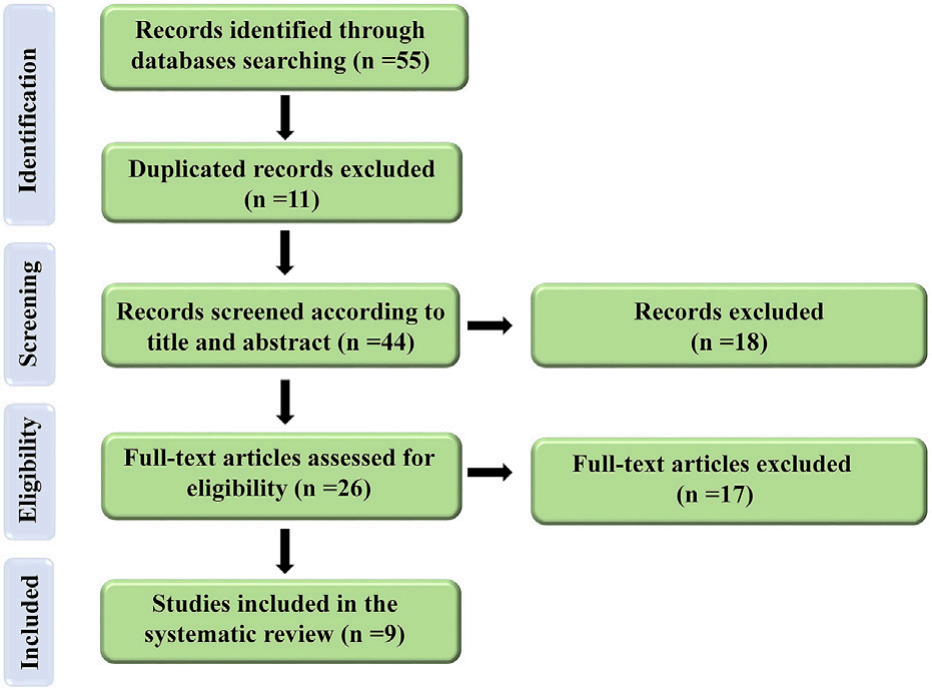
Flow diagram of the selection process for the present study.

A comprehensive search of electronic databases up to March 2022 yielded 47 articles. After excluding duplicated articles (*n* = 11) in the first screening (based on our keywords in the title and abstract), 18 articles were discarded and 18 articles were reviewed in the second screening using our full-text criteria. Finally, nine articles were determined to be eligible for the current investigation. The approach for doing the literature search and screening is depicted in Figure 1.

The information of articles, including the name of the first author and the year of publication, models (tissue) and duration, diabetes induced agent (dosage) and route of administration, outcomes of DN impairment, melatonin dosage, and route of administration/duration of administration, melatonin administration outcomes, were extracted and collected by AH and rechecked by HHA demonstrated in Table 1.

**TABLE 1:** The characteristics of included studies.

Author & year	Models (tissue) & duration	Diabetes induced agent)dosage (& route of administration	Outcomes of diabetic neuropathy impairment	Melatonin dosage & route of administration/duration of administration	Melatonin co-administration outcomes
Babaei et al. (2010)	Diabetic peripheral neuropathy (Rat dorsal root ganglion neurons) & 3 weeks	STZ (50 mg/kg) & ip	↑Pain indices, ↑MDA levels, ↓GPx activity, ↓CAT activity	10 mg/kg/day & ip & 2 weeks after 21st day of diabetes induction	↑Pain indices, ↓MDA levels, ↑GPx activity, ↑CAT activity
Baydas et al. (2003a)	Diabetic neuropathy (Rat hippocampus, cortex, and cerebellum) & 6 weeks	STZ (50 mg/kg) & ip	↑GFAP level, ↑S100B level, ↑MDA levels	10 mg/kg/day & ip & 6 weeks	↓GFAP level, ↓S100B level, ↓MDA levels
Metwally et al. (2018)	Diabetic central Neuropathy (Rat brain, liver, and kidney) & 45 days	STZ (60 mg/kg) & ip	↓CAT, SOD, GPx levels, ↑MDA level, ↑Neurodegeneration	50 mg/kg/day & ip & 72 h prior to diabetes induction and continued daily for 45 days	↑CAT, SOD, GPx levels, ↓MDA level, ↓Neurodegeneration
Negi et al. (2010)	Diabetic Neuropathy (Rat nerves) & 6 weeks	STZ (55 mg/kg) & ip	↓MNCV, ↓Nerve blood flow, ↑MDA level, ↑Peroxynitrite level, ↑IHC score, ↓Hyperalgesia, ↓Allodynia	3 and 10 mg/kg/day & po & 6 weeks after diabetes induction and was continued for 2 weeks	↑MNCV and ↑Nerve blood flow, ↓MDA level, ↓Peroxynitrite level, ↓IHC score with 3 and 10 mg/kg/day, ↑Hyperalgesia and ↑Allodynia with10 mg/kg/day
Negi et al. (2011)	Diabetic Neuropathy (Rat nerves) & 6 weeks	STZ (55 mg/kg) & ip	↑TNF-a and IL-6 Levels, ↑Expression of NF-kB, phosphorylated IkB, iNOS and COX-2 proteins, ↓Expression of Nrf2 and HO-1 proteins, ↑DNA damage	3 and 10 mg/kg/day & po & 6 weeks after diabetes induction and was continued for 2 weeks	↓TNF-a and IL-6 Levels with10 mg/kg/day, ↓Expression of NF-kB, phosphorylated IkB, iNOS and COX-2 proteins, ↑Expression of Nrf2 and HO-1 proteins, ↓DNA damage with 3 and 10 mg/kg/day
Seyit et al. (2016)	Diabetic Neuropathy (Rat tibial nerves) & 4 weeks	STZ (50 mg/kg) & ip	↓MNCV	10 mg/kg/day & po & 4 weeks after diabetes induction and was continued for 2 weeks	↑ MNCV
Magar et al. (2020)	Diabetic Neuropathy (Rat liver and sciatic nerve) & 8 weeks	STZ (45 mg/kg) & ip	↓Escape latency, ↓ MNCV, ↓Hepatic mRNA expression of PGC-1**α** and TFAM, ↑Liver damage, ↑Degeneration of the sciatic nerve	25 and 50 mg/kg/day & po & 8 weeks after diabetes induction and was continued for 4 weeks	↑Escape latency, ↑ MNCV, ↑Hepatic mRNA expression of PGC-1**α** and TFAM, ↓Liver damage, ↓Degeneration of the sciatic nerve
Afifi, (2013)	Diabetic Neuropathy (Rat sciatic nerves) & 6 weeks	STZ (60 mg/kg) & ip	↑Demyelination, ↓Number of total and apparently normal fibers, ↑Number of apparently degenerated fibers	10 mg/kg/day & ip & 6 weeks	↓Demyelination, ↑Number of total and apparently normal fibers, ↓Number of apparently degenerated fibers
Zangiabadi et al. (2011)	Diabetic Neuropathy (Rat sciatic nerves) & 6 weeks	STZ (45 mg/kg) & ip	↓ MNCV, ↑Abnormal myelinated fibers, ↑Axonal degeneration	10 mg/kg/day & ip & 6 weeks	↑ MNCV, ↓Abnormal myelinated fibers, ↓Axonal degeneration

↑, Increase; ↓, Decease; &, and; ip, Intraperitoneal; MDA, Malondialdehyde; GPx, Glutathione peroxidase; CAT, *Catalase*; GFAP, Glial fibrillary acidic protein; S100B, S100 calcium-binding protein B; MNCV, Motor nerve conduction velocity; IHC, Immunohistochemistry; TNF-a, Tumor necrosis factor; IL-6, Interleukin 6; NF-KB, Nuclear factor kappa B; iNOS, Inducible nitric oxide synthase; COX-2, Cyclooxygenase-2; Nrf2, Nuclear factor erythroid 2-related factor 2; HO-1, Heme oxygenase-1; PGC-1**α**, Peroxisome proliferator-activated receptor-gamma coactivator 1- **α**; TFAM, Transcription factor A.

### 3.2 The role of melatonin against biochemical, molecular, histological, and behavioral changes induced by diabetic neuropathy

#### 3.2.1 Biochemical changes

In the present study, biochemical changes are markers for identifying impairments produced by DN. According to the results of this study, DN induces a significant elevation in MDA and peroxynitrite levels as oxidative stress markers and TNF-α and IL-6 levels as inflammation markers compared to the normal group. Meanwhile, melatonin reduced and normalized oxidative stress (Baydas et al., 2003a; Negi et al., 2010; Metwally et al., 2018) and inflammation markers (Negi et al., 2011) compared to the diabetic neuropathic group.

The result of the present study indicated that DN significantly reduced CAT, SOD, and GPx levels as anti-oxidant markers compared to the control group. These anti-oxidant markers were restored to normal levels with melatonin administration (Baydas et al., 2003a; Babaei et al., 2010; Metwally et al., 2018).

#### 3.2.2 Molecular changes

Molecular changes are markers for identifying cell damages produced by DN. Based on the results of this study, DN induces a significant elevation in the expression of NF-KB, phosphorylated IKB, iNOS, and COX-2 proteins as inflammation markers compared to the standard group. Melatonin reduced the expression of NF-KB, phosphorylated IKB, iNOS, and COX-2 proteins compared to the diabetic neuropathic group (Negi et al., 2011).

The result of the present study indicated that DN significantly reduced the expression of Nrf2 and HO-1 proteins as anti-oxidant markers compared to the control group. Melatonin administration restored these anti-oxidant markers to normal levels (Negi et al., 2011).

#### 3.2.3 Histological changes

Histological changes are markers for identifying tissue damages induced by DN. DN induced an increase in GFAP and S100B levels, IHC score, DNA damage, liver damage, neurodegeneration, degeneration of the sciatic nerve, number of apparently degenerated fibers, axonal degeneration, demyelination, abnormal myelinated fibers, as compared to the control group, while treatment with melatonin reversed back these histological changes to normal (Baydas et al., 2003a; Negi et al., 2010; Negi et al., 2011; Zangiabadi et al., 2011; Afifi, 2013; Metwally et al., 2018; Magar et al., 2020).

Moreover, the results of the research indicated that DN decreased nerve blood flow, the number of total and standard fibers, hepatic mRNA expression of PGC-1**α** and TFAM, versus the control group. Whereas melatonin administration increased nerve blood flow, several standard fibers, hepatic mRNA expression of PGC-1**α** and TFAM compared with DN group (Negi et al., 2010; Afifi, 2013; Magar et al., 2020).

#### 3.2.4 Behavioral changes

Behavioral changes are markers for identifying behavioral disorders induced by DN. The evaluation of studies indicated that DN reduced MNCV, hyperalgesia, allodynia, escape latency, and elevated pain indices compared to the normal group. These behavioral changes were restored to normal levels with melatonin administration (Babaei et al., 2010; Negi et al., 2010; Zangiabadi et al., 2011; Seyit et al., 2016; Magar et al., 2020).

## 4 Discussion

**FIGURE 2:**
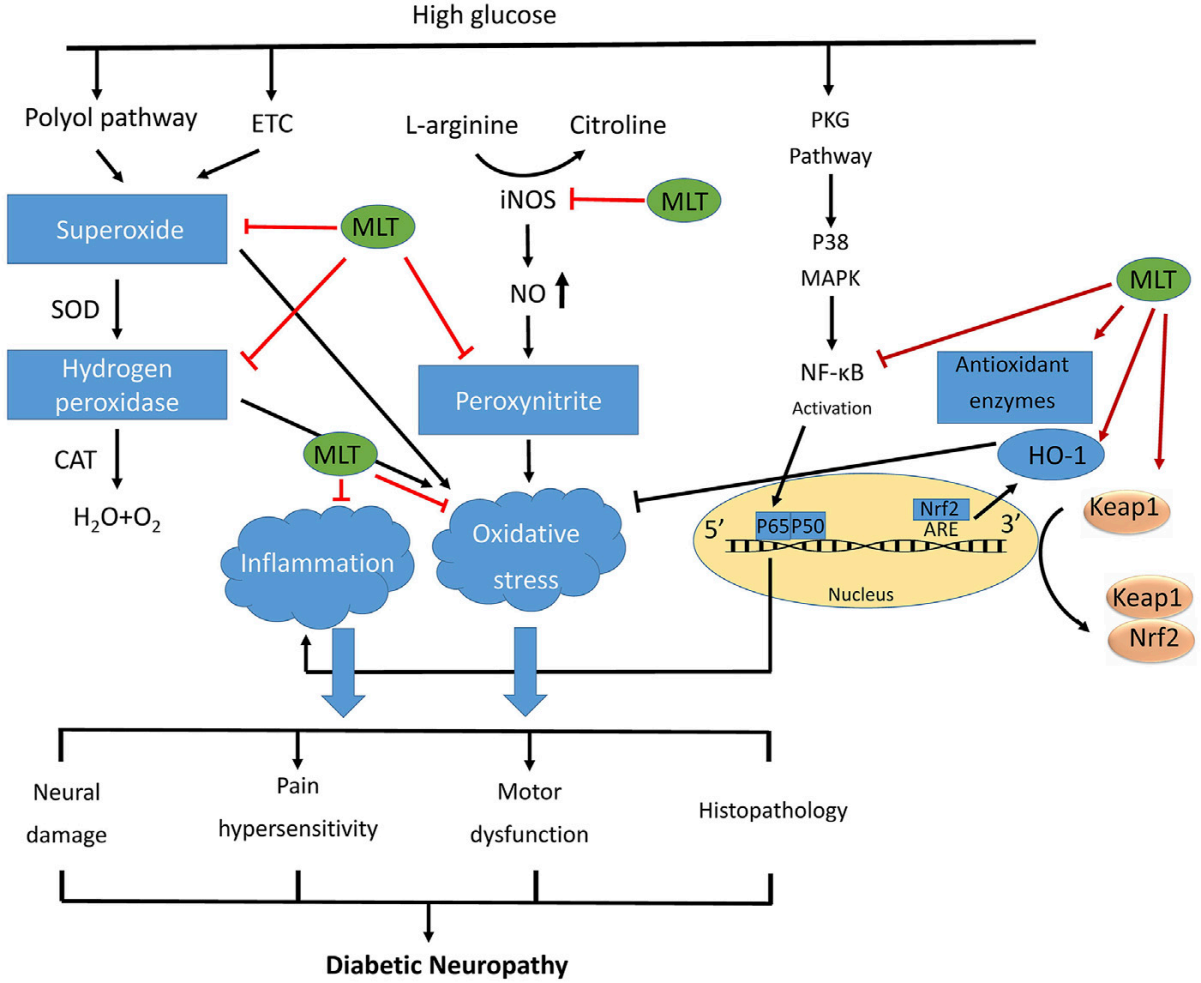
The general mechanisms of diabetic nephropathy.Schematic diagram of the protective function of melatonin against diabetic neuropathy by improving oxidative stress and inflammatory responses. Hyperglycemia activates many metabolic pathways like polyol pathway, and turnover of the mitochondrial energy-production complexes, leading to release of reactive intermediates. High glucose condition activates also PKC pathway, which lead to NF-kB activation. Melatonin inhibits reactive intermediates and NF-kB, and activates Nrf2, and as well as up regulates proteins expression of antioxidant enzymes, resulting in inhibits the oxidative stress and inflammation, and ultimately alleviating the development of diabetic neuropathy. ETC: electron transport chain; PKC: protein kinase c.

This study aimed to review the harmful effects of diabetes on neuron cells/tissue. Additionally, the effects of melatonin administration on these detrimental effects were examined. It is reported melatonin is safe even in extremely high doses, tests on both humans and animals have shown that short-term usage of melatonin is quite safe. There have only been reported relatively minor adverse effects, such as lightheadedness, headaches, nausea, and tiredness. Exogenous melatonin has not been shown in any study to have the potential to cause any major adverse effects (Andersen et al., 2016; Oliveira-Abreu et al., 2021). Table 1 summarizes the data on the effects of DN alone or in combination with melatonin on neuron cells/tissue. Additionally, Figure 2 depicts some of the significant alterations in the neuron cell following diabetes and the effects of melatonin administration on these changes.

Usually, free radicals are created in cells, and their defensive mechanisms neutralize them (Mortezaee et al., 2021). Notably, free radicals increase under oxidative stress due to an imbalance between free radicals and these defensive systems. According to literatures, diabetes raises reactive oxygen species (ROS) in neuron cells/tissue. The ROS assault the cell’s macromolecules, causing the neuron cells/tissue to malfunction (Shiri et al., 2016; Rahimifard et al., 2020).

Oxidative stress is initiated by an increase in free radicals such as superoxide (O_2_^−^), hydroxyl (OH−), singlet oxygen (^1^O_2_), and extra secondary ROS and a reduction in anti-oxidant agent defense (Tipoe et al., 2007). Increased free oxygen radicals could damage cellular carbohydrates, proteins, lipids, and nucleic acids, ultimately resulting in illness (Mathew et al., 2011). It has been demonstrated that diabetes-induced oxidative stress contributes to neuron dysfunction on a molecular level (Chung et al., 2003). Some ROS with a short half-life combines to form more strong free radicals with a long half-life that are more hazardous, including Hydrogen peroxide (H_2_O_2_), HOCl, and OCl^−^ (Boukhenouna et al., 2018). Additionally, H_2_O_2_ can create OH^−^ by the Fenton reaction, which is a highly reactive and poisonous compound (Lyngsie et al., 2018). Anti-oxidant enzymes (SOD, catalase, and GPx) protect tissues and cells from oxidative stress-induced damage by neutralizing free radicals (Rao et al., 2011). Thus, more ROS is produced when anti-oxidant enzymes are depleted. These reactions occasionally result in other hazardous reactive species such as OH^−^ radicals (Beneš et al., 1999; Baeeri et al., 2018). ROS is the rate-limiting step in creating LPO, and so its creation is shown by an increase in the quantities of MDA and other thiobarbituric acid reactive compounds (TBARs) (Princ et al., 1998). Thus, ROS reacts with cell components, impairing cell function (Newsholme et al., 2016). Melatonin is a well-known powerful anti-oxidant with multiple properties that block oxygen-free radicals. Melatonin both directly and indirectly decreases oxidative tissue damage. Melatonin has been demonstrated to act as a direct anti-oxidant, scavenging oxygen free radicals (Najafi et al., 2020a). As a result, it may help to reduce LPO and MDA. Additionally, melatonin has been shown to boost the activity of anti-oxidant enzymes such as SOD, catalase, and GPx by elevation anti-oxidant defenses (Tomás‐Zapico and AJJopr, 2005).

The superoxide dismutase (SOD) enzyme is the body’s first line of defense against superoxide (Ighodaro and OJAjom, 2018). SOD converts O_2_^−^ free radicals to H_2_O_2_, and oxygen molecule, which is then eliminated by catalase, and GPx (Kurutas, 2016).

Catalase is the enzyme that converts H_2_O_2_ to H_2_O and O_2_ (Aksoy et al., 2005). GSH is a tripeptide critical for the cell’s protection against free radicals (Forman et al., 2009). GSH reacts with oxygen free radicals, decreasing their concentrations; in this case, neuronal GSH levels drop during oxidative stress due to its oxidation to glutathione disulfide (GSSG) (Dringen et al., 2000). Additionally, the glutathione reductase (GR) enzyme reduces GSSG to GSH by the oxidation of NADPH to NADP^+^ (Yang et al., 2006). Moreover, diabetes induced DNA damage in a neuron is ameliorated by melatonin (Negi et al., 2011).

The importance of oxidative stress in the pathogenesis of several neurodegenerative illnesses has been well recognized. It has been proved that increasing HO-1 *via* genetic or pharmacological modification confers stress tolerance and neuroprotection (Calabrese et al., 2008).

The protective function has been demonstrated to be due to the manufacture of HO-1 activity’s end products, as shown by the use of ferulic acid, which increases bilirubin and CO production in response to -amyloid-induced oxidative stress (Joshi et al., 2006). According to other research, CO suppresses AMP-activated protein kinase (AMPK) activity, which has been associated with -amyloid-induced damage. Experiments on animal models have revealed that HO-1 protects neurons against oxidative damage (Wen et al., 2020; Engin, 2021).

In eukaryotic organisms, the nuclear factor erythroid 2-related factor 2 (Nrf2) is a transcription factor that regulates cellular redox equilibrium (Dai et al., 2020). It plays a serious function in phase II detoxification enzymes and stress proteins, *via* modifying the expression of genes, containing anti-oxidant/electrophile response elements (Kim et al., 2010). Given this crucial cytoprotective regulatory role for genes and proteins, the Nrf2/HO-1 axis in neuroprotection is considered as the focus research in recent years under normal and pathological conditions (Espada et al., 2010). It is also involved in neural resistance to oxidative stress and glutamate-induced excitotoxicity (Brasil et al., 2021). It is known that astrocytes protect neurons from damage by regulating Nrf2 signaling, but little is known about how astrocytes regulate neuronal Nrf2 signaling (Habas et al., 2013). This study shows that increased neuronal activity triggers the release of soluble substances that activate the astrocytic Nrf2 pathway *via* group I metabotropic glutamate receptors and intracellular Ca^2+^ (Ishii et al., 2019). The neuron–astrocyte tripartite synapse regulates endogenous anti-oxidant signaling by matching astrocyte neuroprotective capacity to neighboring neuronal synaptic activity (Sorrentino et al., 2019). The results show that DN reduces HO-1 and NRF2 expression, which melatonin reverses (Negi et al., 2011). The possible mechanism is mediated through the anti-oxidant role of melatonin.

GFAP and S100B are either trophic or poisonous in high quantities (Rothermundt et al., 2003). Overproduction of neuronal and glial markers results in neuronal and glial death, while low levels operate as neurotrophic factors, which may be significant during growth and nerve redevelopment (Mirsky et al., 2008). When untreated diabetes with extremely high glucose levels persists, tissue damage is noticed, ascribed to oxidative injury caused by forming free radicals (Giacco and Brownlee, 2010). One of the effects of this type of damage is a depleted supply of the cellular anti-oxidant GSH. Diabetes may result in glial sensitivity due to a weakened defense system against free radicals and increased glutamatergic activity (Uttara et al., 2009). Astrocytes’ increased susceptibility to oxidative stress due to diabetes increases their vulnerability to harm. Injuries to neurons and glia increase GFAP and S100B production (Baydas et al., 2005). Consistent with the current findings, we recently proposed that glial reactivity was intimately tied to the oxidative environment (Andersen, 2004). These free radicals damage macromolecules within the neuron and glial cells, resulting in protein structural and functional alterations (Kumar et al., 2012). GFAP and S100B are produced by glial cells in response to oxidative stress (Baydas et al., 2003b). The present study demonstrated that GFAP, and S100B levels elevated in DN and melatonin through its polytrophic effect reduced GFAP and S100B levels (Baydas et al., 2003b).

The inflammation pathway is a biological reaction to potentially damaging motivations involving various signaling pathways (Chen et al., 2017). Neutrophil cells are the primary inflammatory. Increased pro-inflammatory molecules such as chemokines, cytokines, and others worsen this reaction (Shanmugam et al., 2010). Previous research has established that neuron damage caused by diabetes is associated with inflammation (Muriach et al., 2014). NF-κB is a critical component of the inflammatory response (Liu et al., 2017). Stimulating the transcription factor IκB, NF-κB activates the genes encoding pro-inflammatory cytokines, including TNF-α, and improves the deadly effects of DN (Negi et al., 2011; Najafi et al., 2020b). Additionally, these DN promote the expression of chemokines and pro-inflammatory cytokines, for example, IL-1, IL-6, and TNF-α (Negi et al., 2011). These variables influence the extent to which neutrophils infiltrate the damaged location (Puhl and Steffens, 2019). Melatonin may have anti-inflammatory properties, as evidenced by numerous research (Farez et al., 2016). Possible anti-inflammatory processes include decreasing free radicals, activating NF-κB, down-regulation of chemotaxis, and pro-inflammatory cytokines (IL-1, IL-6, and TNF-α) of inflammation cells (Sarkar and Fisher, 2006).

Sleep patterns are linked to diabetes through metabolic syndrome and insulin resistance, and the link between disrupted sleep and diabetes is likely driven by a lack in melatonin (Wajid et al., 2020). Insulin resistance and metabolic syndrome are both associated to obesity (Anderson et al., 2001). In point of fact, people with type 2 diabetes who slept less had higher 24-h urine protein and albumin excretion levels, which are indications of more severe DN (Xiang et al., 2016). In addition, diabetes-related hyperglycemia causes a decrease in the production of melatonin, which makes it more difficult to sleep and exacerbates metabolic medical disorders (Espino et al., 2011).

The expression of the MT1 and MT2 receptors has been found in the pancreatic tissue of both humans and rodents (Nagorny et al., 2011). According to some publications, the MT1 receptor is more prevalent in alpha cells, whilst the MT2 receptor is only seen in beta cells. Even though an identical level of transcript for each was reported in alpha and beta cells, high-throughput investigation of mRNA abundance using RNA sequencing has established the presence of MT1 and MT2 in human islets (Ramracheya et al., 2008; Forrestel et al., 2017). Melatonin is responsible for regulating a wide variety of cellular processes and gene expression within the islets of the pancreas (Mulder et al., 2009). Nearly simultaneously with the finding that this hormone had “insulin-like” activity came the realization that melatonin has the ability to induce sleep through its sedative properties. In subsequent research, the effects of melatonin on glucose homeostasis were investigated and characterized (Garaulet et al., 2020; Abdulwahab et al., 2021).

In general, based on the non-clinical findings of this study, melatonin, through various mechanisms such as oxidative stress and inflammation by affecting different molecules, including MDA, GPx, CAT, SOD, TNF-α, IL-6, NF-kB, IkB, iNOS, COX-2, Nrf-2, and HO-1 leads to the reduction of neuropathy damage induced by diabetes and the pathological damage. It should be noted that these results are from non-clinical studies and for clinical studies, more studies on different models and more paths are needed.

## 5 Conclusion

In conclusion, this study demonstrates that neuropathy is a type of harm caused by diabetes. Diabetes causes neuropathy by generating toxic free reactive species, initiating inflammatory pathways, and causing histological injury. Melatonin administration during DN reduces the risk of neuropathy by reducing oxidative stress, inhibiting the inflammatory process, and reducing histological damage. As a result of the current study’s findings, treatment with melatonin may result in a more tolerable neuropathy for patients.

## Data Availability

The original contributions presented in the study are included in the article/Supplementary Material; further inquiries can be directed to the corresponding authors.
